# A Comprehensive Analysis of the Transcriptomes of *Marssonina brunnea* and Infected Poplar Leaves to Capture Vital Events in Host-Pathogen Interactions

**DOI:** 10.1371/journal.pone.0134246

**Published:** 2015-07-29

**Authors:** Chengwen Chen, Ye Yao, Liang Zhang, Minjie Xu, Jianping Jiang, Tonghai Dou, Wei Lin, Guoping Zhao, Minren Huang, Yan Zhou

**Affiliations:** 1 State Key Laboratory of Genetic Engineering, School of Life Sciences, Fudan University, Shanghai, People’s Republic of China; 2 Shanghai-MOST Key Laboratory of Health and Disease Genomics, Chinese National Human Genome Center at Shanghai, Shanghai, People's Republic of China; 3 Shanghai Jiao Tong University School of Medicine, Shanghai, People's Republic of China; 4 Center for Computational Systems Biology and School of Mathematical Sciences, Fudan University, Shanghai, People’s Republic of China; 5 Jiangsu Key Laboratory for Poplar Germplasm Enhancement and Variety Improvement, Nanjing Forestry University, Nanjing, People’s Republic of China; Fujian Agriculture and Forestry University, CHINA

## Abstract

**Background:**

Understanding host-pathogen interaction mechanisms helps to elucidate the entire infection process and focus on important events, and it is a promising approach for improvement of disease control and selection of treatment strategy. Time-course host-pathogen transcriptome analyses and network inference have been applied to unravel the direct or indirect relationships of gene expression alterations. However, time series analyses can suffer from absent time points due to technical problems such as RNA degradation, which limits the application of algorithms that require strict sequential sampling. Here, we introduce an efficient method using independence test to infer an independent network that is exclusively concerned with the frequency of gene expression changes.

**Results:**

Highly resistant NL895 poplar leaves and weakly resistant NL214 leaves were infected with highly active and weakly active *Marssonina brunnea*, respectively, and were harvested at different time points. The independent network inference illustrated the top 1,000 vital fungus-poplar relationships, which contained 768 fungal genes and 54 poplar genes. These genes could be classified into three categories: a fungal gene surrounded by many poplar genes; a poplar gene connected to many fungal genes; and other genes (possessing low degrees of connectivity). Notably, the fungal gene M6_08342 (a metalloprotease) was connected to 10 poplar genes, particularly including two disease-resistance genes. These core genes, which are surrounded by other genes, may be of particular importance in complicated infection processes and worthy of further investigation.

**Conclusions:**

We provide a clear framework of the interaction network and identify a number of candidate key effectors in this process, which might assist in functional tests, resistant clone selection, and disease control in the future.

## Introduction

Host-pathogen interactions and co-evolution often involve complicated invasion and defense mechanisms and may exhibit a sophisticated network at the molecular level [1,2]. Understanding these underlying interaction networks helps to elucidate the entire infection process and focus on certain vital events, which is a promising approach for improvement of disease control and selection of treatment strategy [3].

In general, simultaneously investigating the transcriptional profiles of a host and a pathogen in time course experiments is an efficient method of deciphering important changes and correlating each change with the response of its counterpart. However, traditional microarray technologies have the disadvantage of utilizing mixed RNA samples (host and pathogen) or applying a compromised method that extracts RNA from separate parts using different protocols, a process that inevitably increases noise [4,5]. The emergence of high-throughput parallel sequencing technology (RNA-seq), a species-independent platform, provides us with an appropriate solution [6]. Based on the known genomes of hosts and pathogens, expression profiles for the host and pathogen can be accurately constructed from a mixed RNA sample [7,8]. Tierney et al. successfully quantified *C*. *albicans* and *M*. *musculus* gene expression dynamics using simultaneous RNA-seq and predicted novel interactions [8].

Reverse engineering techniques are often used to predict unknown networks based on gene expression data[9], and the functional identification of target genes is one of the major objectives of network analyses using-omics data. A number of data-mining approaches, such as Pearson’s correlation, principal component analysis (PCA), and independent component analysis (ICA), have been introduced or established to unravel the direct or indirect relationships of genes by inferring a biological topology network composed of nodes and edges, where the nodes represent interesting genes and the edges show the relationships[10,11]. Gene co-expression analysis (correlation analysis) based on the so-called ‘guilt-by-association’ principle is frequently used in transcriptome data modeling to identify target genes[12]. The success of this approach has been summarized in an article by Tohge and Fernie; however, it is important to note that candidates obtained this way require a “fair trial” because assuming “guilt” is dangerous, as summarized by Usadel et al.[13]. Furthermore, whole-genome expression datasets obtained from the visualization of complex, temporally, and/or spatially resolved experiments help find “the meaning within the noise”[14]. Nevertheless, time series analyses can suffer from missing data or bias at certain time points due to technical problems such as RNA degradation, which limits the application of algorithms requiring strict sequential sampling.

In this study, we sought to perform a comprehensive analysis of the transcriptomes of a host and a pathogen using RNA-seq data from a time-course infection experiment of poplar leaf and *Marssonina brunnea*. *M*. *brunnea*, a filamentous fungus with a relatively narrow host range, is a causal pathogen of Marssonina leaf spot, which devastates poplar plantations by defoliating susceptible trees and severely reducing the growth and productivity of hybrid poplars[15,16]. To date, few fungicides have been available for controlling Marssonina leaf spot; thus, the most promising counter-measure is to plant poplar varieties that are resistant or tolerant to *M*. *brunnea* [3]. In a preceding work, we completed an *M*. *brunnea* genome sequencing project and simultaneously successfully investigated the host-pathogen transcriptome using RNA-seq[17]. An expression profile was generated from a mixed RNA sample extracted from an infected leaf of poplar (clone NL895) that is highly resistant to *M*. *brunnea*. Based on our poplar-fungus infection model, in this study, we performed a comprehensive time series analysis on host-pathogen transcriptomes to dissect the regulatory network underlying the transcriptional response to the infection. Furthermore, a specific independent test strategy was introduced to construct an interspecies topology network, which indirectly connected the transcriptome of the host and pathogen and showed more details of the interaction.

## Materials and Methods

### Strains

*Marssonina brunnea* f. sp. *multigermtubi*, which infects *Populus* species from Sections *Aigeiros* and *Tacamahaca*, was obtained from the eastern region of China. This pathogen has been studied in our laboratory for approximately 30 years[16]. The poplar clone NL895 is highly resistant to *M*. *brunnea* f. sp. *multigermtubi* and is one of the most important commercial planting clones in China. In contrast, poplar clone NL214 is highly susceptible (weakly resistant) to the disease.

### Infection

Cuttings of clones NL895 (*P*. *euramericana* CL “NL895”) and NL214 were cultured in a greenhouse at 22°C with a 12-hour photoperiod until the cuttings were approximately 0.5–1 m high and had 10 to 20 fully expanded leaves. Five or six fully expanded leaves were collected and placed on 2% agar sterile culture plates with the abaxial surface of the leaf facing upward. Conidia of *M*. *brunnea f*. sp. *multigermtubi* were suspended in sterile water, and the suspension was adjusted to 80,000 spores/ml and sprayed onto the abaxial surface of the poplar leaves. The treated leaves were incubated in an illuminated incubator under 100% relative humidity (RH) at 22°C with a 12-hour photoperiod and were then harvested at 6, 12, 24, 48, 72, and 96 h post-inoculation. All leaves were immediately frozen in liquid nitrogen and stored at −70°C.

### RNA-seq

All five or six *M*. *brunnea*-infected leaves derived from the same condition (treatment, line and time point) were homogenized using Bertin Precellys 24 (plus beads), and then the suspension was mixed together as a sample. No biological replications were performed in subsequent sequencing part. RNA was extracted using the TRIzol reagent according to the manufacturer’s instructions (Invitrogen, Carlsbad, CA, USA). Genomic DNA was removed by DNase I (TaKaRa, Japan), and libraries were constructed using the Illumina standard kit (TruSeqTM DNA Sample Prep Kit), as described in the manufacturer’s protocol. All sequencing was performed using an Illumina HiSeq 2000 (Illumina Inc., San Diego, CA, USA). All the RNAseq data (fastq format) were submitted to NCBI SRA database (SRP042102, http://www.ncbi.nlm.nih.gov/sra/?term=SRP042102).

The RNA-seq reads were mapped onto the genomes of *M*. *brunnea* and *Populus trichocarpa* separately (v2.10, http://genome.jgi-psf.org/poplar/poplar.home.html) using TopHat (v2.0.8, http://tophat.cbcb.umd.edu/, allowing 2 mismatches per read as the default). The Samtools rmdup function was used to eliminate the bias introduced by PCR amplification, and paired reads that mapped to different chromosomes were discarded. Shared mapped reads (that could be mapped to the fungus and poplar genomes) were excluded from our gene expression profile analysis. The numerical measure of the mapped fragments (a fragment means a paired read) was used to evaluate the relative expression level of a certain gene. The value for a multi-mapped read, one that mapped to multiple positions, was divided by the number of positions. For instance, each position of a read that mapped to 10 positions will count as 0.1. In an individual library, the gene expression measure was normalized using the total number of mapped fragments. The reads count normalization was performed on each organism separately. Differentially expressed genes (DEGs) between two libraries were identified using Fisher’s exact test. Principal component analysis (PCA) was used to explore the overall expression pattern (poplar genes) of these samples during infection. All perl scripts and processed data can be downloaded from the website (http://homepage.fudan.edu.cn/zhouyan/interactions/).

### Independent network inference

Various relevance measures have been used to infer relationships between two genes, from simple correlation measures to biologically motivated relevance measures[14]. Here, we introduce another efficient method using independence test to infer an independent network.

Briefly, given a set of n genes, G = {g1, g2,…, gn}, with D as a set of observations at different time points (k) on the expression profiles of the total genes, the relevance between two genes, gi and gj, could be evaluated using their profiles at different time points: [gi1, gi2,…, gik] and [gj1, gj2, …, gjk]. For every two observations, we considered the differential expression (P<0.01, ratio>2 or <0.5) and classified the varieties of gene expression into nine possible conditions, which resulted from the combination of three trends (up-regulation ↑, down-regulation ↓, and unchanged-) of the expression of the two genes (S1 Fig). The frequency for every condition was calculated and assigned to the appropriate cells of a 3*3 grid. To decrease the influence of inactive genes (e.g., stably expressed genes), we excluded the gi(-)gj(-) condition and split the 3*3 grid into four fourfold tables; independence was assessed using these tables (Fisher’s exact test) [18]. A smaller p value reflected a higher relevance (lower independence); therefore, we defined the smallest p value of the four tables as the pij relevance [19]. All relevance p-values were calculated for the genes. The paired genes were sorted by their relevance p-value in ascending order, and the top 1,000 were imported into the Cytoscape software to perform a visual network analysis. Independence tests were performed using perl script.

### Effector prediction

The secretome was used to predict the candidate effectors of the fungus using domain analysis. The secreted proteins were identified using the TargetP [20] and TMHMM [21] tools and the criteria that proteins with extracellular signals had 0 or 1 transmembrane domains but no GPI (glycosyl-phosphatidylinositol) anchor domains. The secreted proteins containing an effector-specific domain significantly enriched in known effectors [22] were considered to be candidate effectors.

## Results

### Overview of the host-pathogen infection transcriptomes

NL895 and NL214 poplar leaves were infected by *M*. *brunnea* and harvested over a series of time points. Comprehensive gene expression profiles of the pathogen and host were generated using RNA-seq technology. Depending on the proliferative and infectious capacities of the fungus, we classified the samples into two groups (Table 1): highly active (containing two subgroups, 214/highly active and 895/highly active) and weakly active (214/weakly active and 895/weakly active). The leaves infected with highly active fungi showed many obvious Marssonina leaf spot several days later (S2 Fig), whereas those infected with weakly active fungi did not (or show sporadic spots occasionally). Increasing the initial inoculum of weakly active fungi did not significantly increase the number of disease spots. In addition, the biomass of weakly active fungi rapidly decreased during infection, which was different from gradual proliferation of highly active fungi. The initial spores were adjusted to a level at which the number of leaf spot reached the peak at later stage (NL 895 leaves infected with highly active fungi), and this inoculum concentration was also used in other experiments. In this study, we did not set zero time point, because it was hard to detect the expression of fungal genes in mixed samples using realtime PCR, due to the small number of spores sprayed on the leaves. Additionally, the most of fungi were still in the form of spore at zero time point, which was different from the free living status on the leaves during infection. Thus, we chose “12h” as the first time point, “48h” as intermediate, and “96h” as last for highly active fungi according to their infection period, and harvested the infected leaves at these time point for RNAseq. Because the weakly active fungi only survived a shorter time on the surfaces of the leaves, we bring forward the first time point to 6h (followed by 24h and 72h). Certain samples were excluded for insufficient RNA quality (Agilent 2100 quality control), and 126,869,991×2 paired-end reads were generated from the nine libraries (Table 1). S1 Table showed the Statistics information for mapped reads and detected genes. The 214 24-h library was sequenced at a higher depth (4.5 gigabasepairs, Gbps), whereas the data size of the other libraries was greater than 2 Gbps.

**Table 1:** Summary of the available infected samples in the time-course experiment.

	Highly active fungi	Weakly active fungi
**NL214 (Weakly resistant)**	48 h (2.8 G^a^)	24 h (4.5 G)
	96 h (3.0 G)	
**NL895 (Highly resistant)**	12 h (2.9 G)	6 h (2.6 G)
	48 h (3.0 G)	24 h (2.3 G)
	96 h (3.6 G)	72 h (2.6 G)

^a^1 G = 5 million paired-end reads×100 bp.

**Fig 1:**
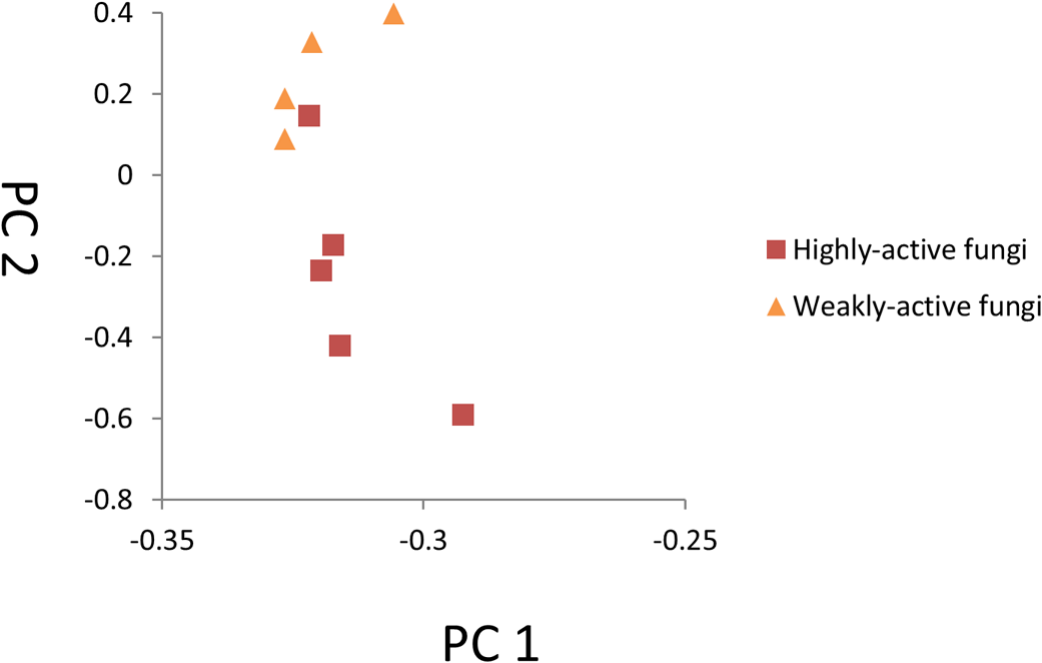
Principal component analysis (PCA) of poplar genes during infection. The graph illustrates the distribution of each sample in the space of the first two principal components (PCs). The samples infected with weakly active fungi are clustered together, whereas the distribution of the highly active group is relatively scattered.

In PCA analysis, PC1 and PC2 explained 95% of the variations in gene expression and represented the differences between these libraries (Fig 1). Notably, the samples infected with weakly active fungi were clustered, whereas the distribution of the highly active group was relatively scattered, which suggested large changes at the gene expression level during infection by highly active fungi.

### The mapped fragments and detected genes exhibited obvious differences among the time-course samples

By examining the proportion of the mapped fragments of poplar leaves to those of *M*. *brunnea* (defined as the P:M proportion), we found that the leaves infected with highly active fungi often had a higher proportion of fungal-related fragments than their counterparts that were infected with weakly active fungi (S3 Fig). The time point selection had little influence on the P:M proportion in the weakly active group; however, in the highly active group, the proportion of fungal-related fragments increased in the NL895 96 h samples compared with the 12 h and 48 h samples. This finding might reflect the faster proliferation of the highly active fungi during infection.

We investigated the number of detected genes for poplar and *M*. *brunnea* using a criterion that the mapped fragments were not less than 2 (S1 Table). Of the 10,040 predicted genes in *M*. *brunnea* and the 41,335 predicted genes in *Populus*, 6,898 (69%) and 30,977 (75%) were identified in the nine samples, respectively, suggesting a relatively high coverage of the transcriptomes. The sequencing depth varied from 2.3 Gbps to 4.5 Gbps among these samples. Although higher depth was always suggested, now that the leaves took a high proportion in the mixed sample, it was limited to improve the coverage of transcriptomes of fungi by increasing depth. A higher sequencing depth (NL214 24 h library) did not significantly increase the number of detected genes. When focusing on the NL 895 clones, we found that the number of detected poplar genes was similar between the highly active (12, 48, and 96 h) and weakly active groups (6, 24, and 72 h), sharing 27,345 expressed genes (S4 Fig). However, more fungal genes were detected in the highly active group than the weakly active group, with 2,123 overlapping genes. This might be also a result of a more abundant RNA level in the highly active *M*. *brunnea* sample due to the higher fungus proliferation.

### *M*. *brunnea* gene expression profile analysis and the characteristics of highly and weakly active fungi

To study the infection-associated differentially expressed genes (DEGs), the transcriptomes were analyzed and compared to the reference transcriptome at the early stage of infection (time point 6 h for weakly active fungi and 12 h for highly active fungi).

**Fig 2:**
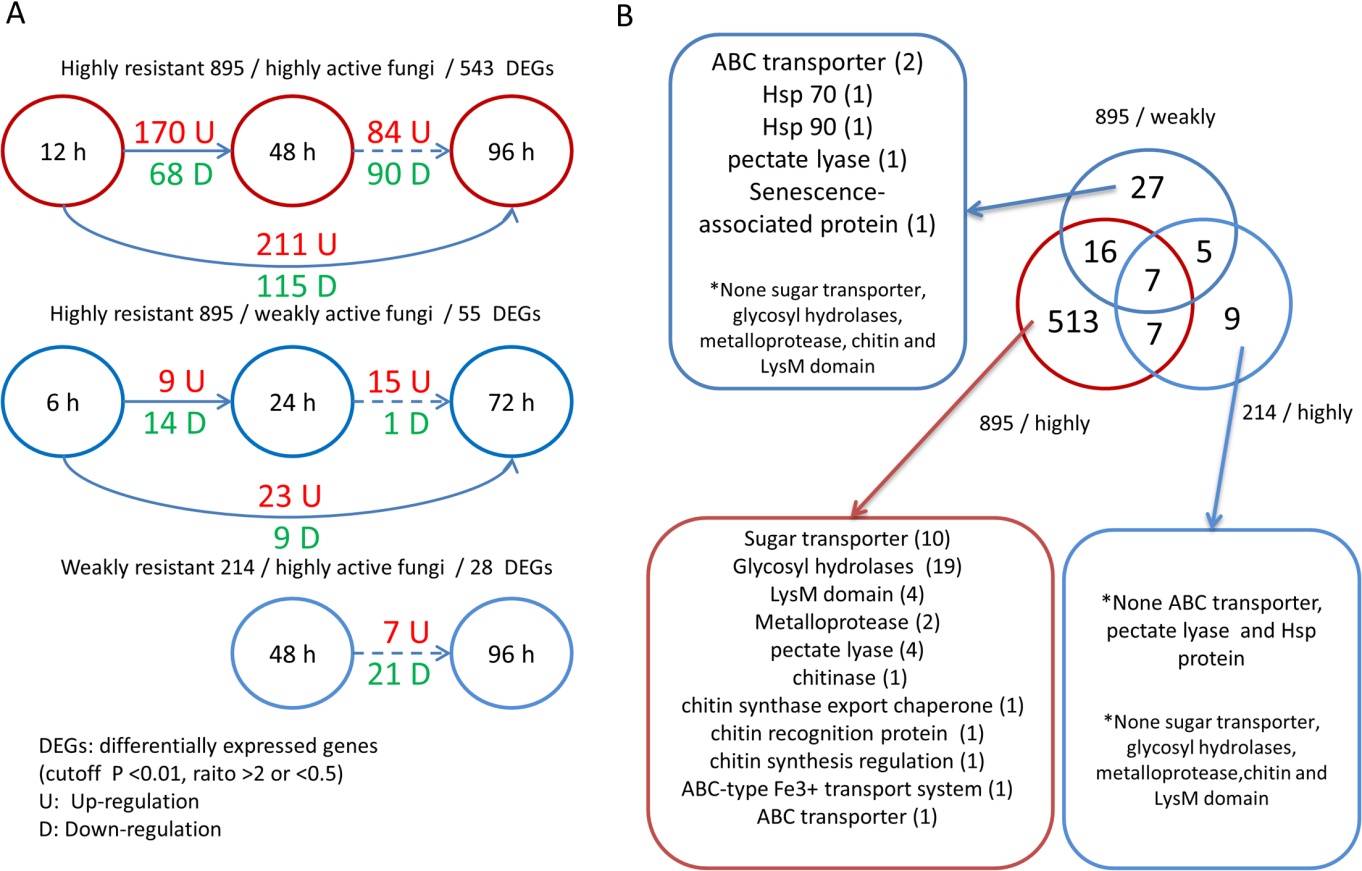
The characteristics of 584 DEGs in dynamic transcriptome analysis suggested intense rivalry in the highly resistant 895/highly active fungus group. (A) The differentially expressed fungal genes between different time points in three groups. (B) The overlapping and specific DEGs among the three groups. The numbers in the brackets represent the number of DEGs belonging to the annotation.

In the 895/highly active group, 238 *M*. *brunnea* genes displayed different levels of expression from 12 to 48 h (P<0.01, ratio >2 or <0.5), and 326 genes did so from 12 to 96 h (Fig 2A). However, the number of DEGs in the 895/weakly active group decreased to 23 (from 6 to 24 h) and 32 (from 6 to 72 h). Furthermore, the 214/highly active group had only two samples and 28 DEGs (48 vs. 96 h), which was far less than the comparison between 48 and 96 h for the 895/highly active group. Lastly, in the dynamic transcriptome analysis, we obtained 584 fungal DEGs, most of which originated from the 895/highly active group, which might suggest an intense rivalry between highly resistant poplar leaves and highly active fungi.

Of the 513 895/highly group-specific DEGs, 301 were up-regulated, 188 were down-regulated, and 24 exhibited fluctuating trends during the infection. According to the annotation, several transporter genes, in particular, 10 sugar transporter genes that might play important roles in host-pathogen sugar intake competition, were overexpressed in the later stage of infection (Fig 2B). Moreover, 19 glycosyl hydrolases, which are related to host cell wall degradation, were significantly differentially expressed. The expression levels of two metalloproteases and four pectate lyases were also altered during the infection. These enzymes are critical for pathogen invasion and cause damage by digesting structural components of the host cell [23]. Three DEGs were from the LysM family, which functions as effectors to suppress plant basal immunity during plant colonization. In addition, the gene expression of a chitin-associated enzyme and ABC transporter genes was also significantly altered. In contrast, no sugar transporter, glycosyl hydrolase, metalloprotease, chitin, or LysM domain genes were found in the 895/weakly group-specific DEGs, except for two ABC transporter genes. In particular, this group contained two Hsp proteins (Hsp 70 and Hsp 90) and a senescence-associated protein. Interestingly, none of the preceding types of genes were found in the 214/highly active group. The characteristics of these group-specific DEGs implied varied mechanisms and strategies in the fungus-poplar interactions of highly and weakly active fungi when fighting against a powerful or weak rival.

### Populus transcriptomes revealed specific expression patterns of disease-resistance genes during infection

**Fig 3:**
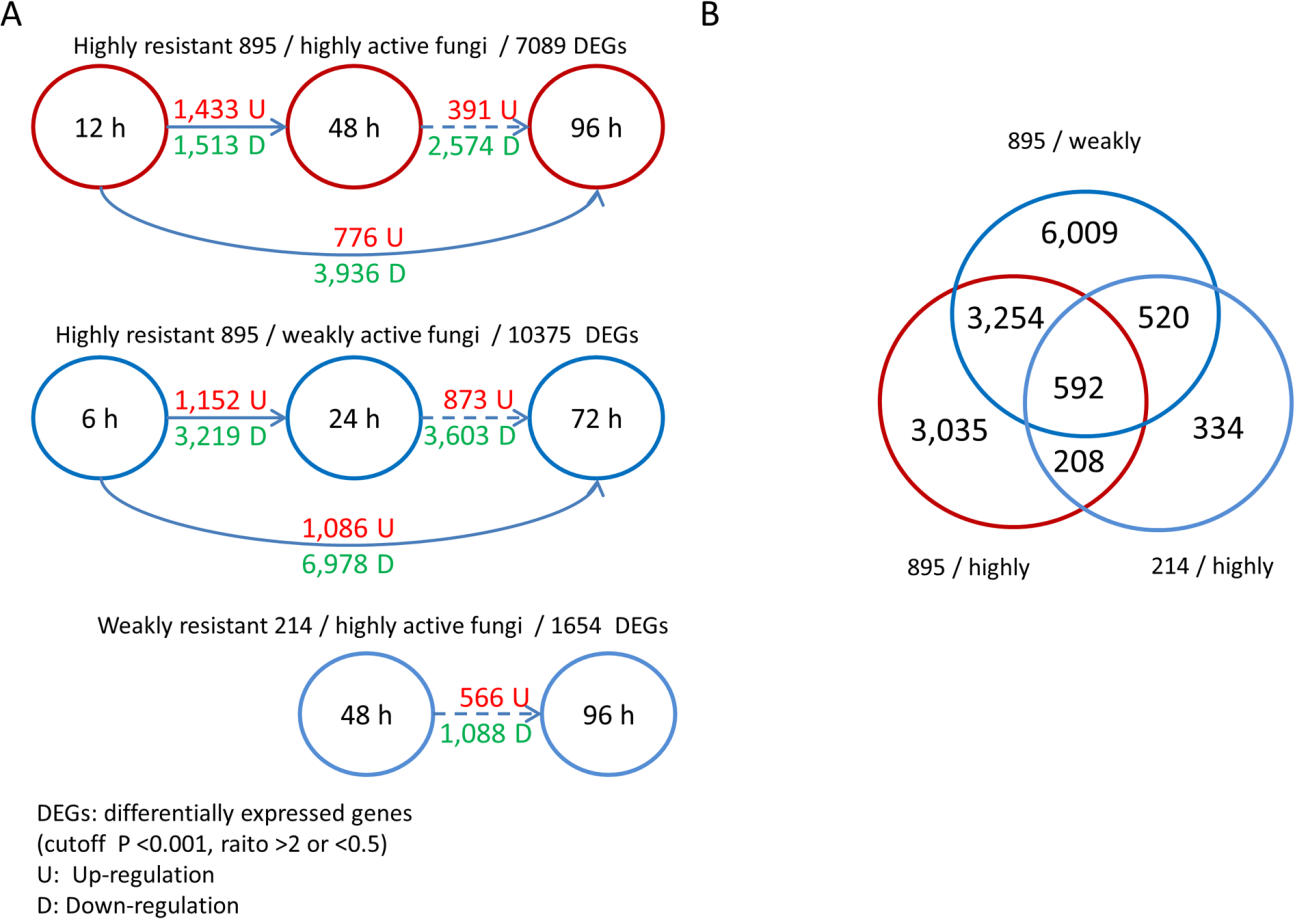
More poplar genes were up-regulated in the highly resistant 895/highly active fungus group at the early stage. (A) The differentially expressed poplar genes between different time points in three groups. (B) The overlapping DEGs among the three groups.

We adopted the same comparison patterns for the poplar transcriptome analysis as we did for the fungal analysis. In the 895/highly active group, 2,946 poplar genes displayed different levels of expression from 12 to 48 h (P<0.001 and ratio >2 or <0.5), and 4,712 genes did so from 12 to 96 h (Fig 3A). Unexpectedly, more genes were significantly differentially expressed in the 895/weakly active group (4,371 DEGs from 6 to 24 h; 8,064 DEGs from 6 to 72 h), and most were down-regulated. However, compared with the 895/weakly active group, more poplar genes were up-regulated in the 895/highly active group at the early stage (from 12 to 48 h), which reflects the host’s powerful response to pathogenic invasion. The overlapping genes (3,846) only constituted a small portion of these two groups, which might also suggest that different interaction mechanisms were triggered when a highly active or weakly active pathogen infected the plant (Fig 3B). The 214/highly active group only had two samples and 1,654 DEGs (48 vs. 96 h), most of which overlapped with the two other groups.

**Fig 4:**
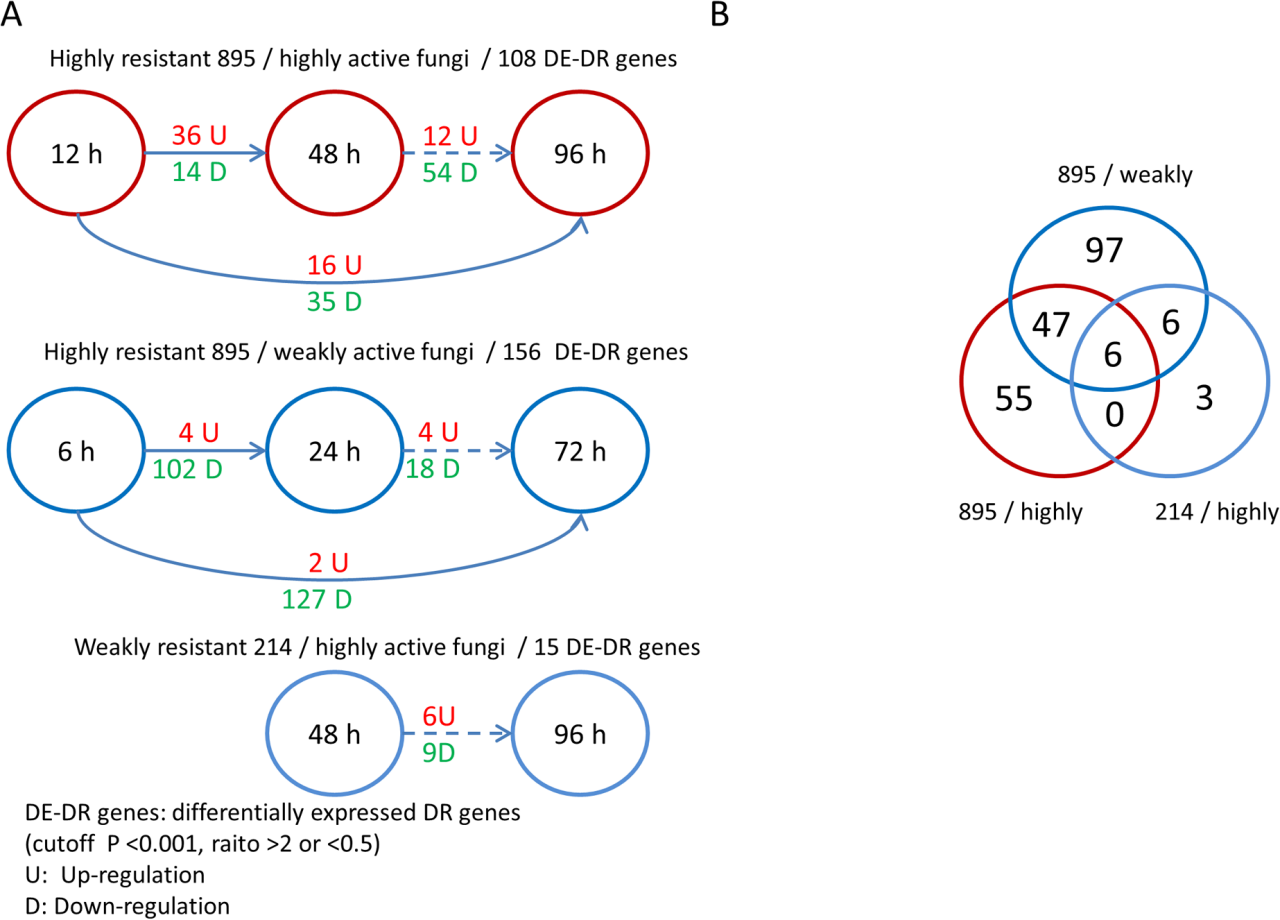
More poplar DR genes were up-regulated in the highly resistant 895/highly active fungus group during infection (especially during the early stage). (A) The differentially expressed poplar disease-resistant genes between different time points in three groups. (B) The overlapping DE-DR genes among the three groups.

We concentrated on the expression changes in disease-resistance (DR) genes, which include a variety of intracellular receptors that could induce effector-triggered immunity (ETI)[24]. Of the 41,335 predicted genes in *Populus*, 761 were defined as DR genes. Fig 4 demonstrates the alterations in DR gene expression of all three groups. The number of significantly differentially expressed disease-resistance genes (DE-DR genes) was greater in the 895/weakly active group than that in the 895/highly active and 214/highly active groups, but most of these DR genes (95%) were down-regulated in the 895/weakly active group. Conversely, in the 895/highly active group, the gene expression levels of half of the DE-DR genes increased from the early to late stage. In particular, from 12 to 48 h, the up-regulated DE-DR genes far exceeded the down-regulated genes (Fig 4A). When suffering from infection caused by highly active fungi, more DR genes might be activated (up-regulated) to induce the immune response in highly resistant 895 clones. However, weakly active fungi might be unable to trigger many immune responses due to their different influence on the host. The 214/highly active group only had 15 DE-DR genes (48 vs. 96 h), and most overlapped with other two groups. Otherwise, the 895/weakly active group only shared 34% of DE-DR genes with the 895/highly active group (Fig 4B). Of the 761 DR genes, only 214 showed significant expression changes during the entire infection. We found that, of the DR genes, the DE-DR genes mainly originated from NB-ARC domain-containing disease-resistance proteins and TIR-NBS-LRR class family disease-resistance proteins (S2 Table).

### Independent network analysis shows vital relationships between host and pathogen

To elucidate gene-gene interactions between the pathogen and host, independent network inference was performed between the fungal and poplar genes. To increase the sample size, the RNA-seq data of 895 poplar leaves that were infected with highly active fungi in our previous work were also included in this analysis. Infected samples were selected, and the time of comparison between these samples was C^2^_10_ = 10*9/2 = 45. The assessment criteria of differential expression was P<0.05, a ratio >2 or <0.5 for fungi and P<0.01, ratio>2 or <0.5 for poplar. The relevance for fungus-poplar (F-P) combinations (6,898 detected fungal genes * 30,977 detected poplar genes = 27,433,346 relationships in total) was evaluated.

**Fig 5:**
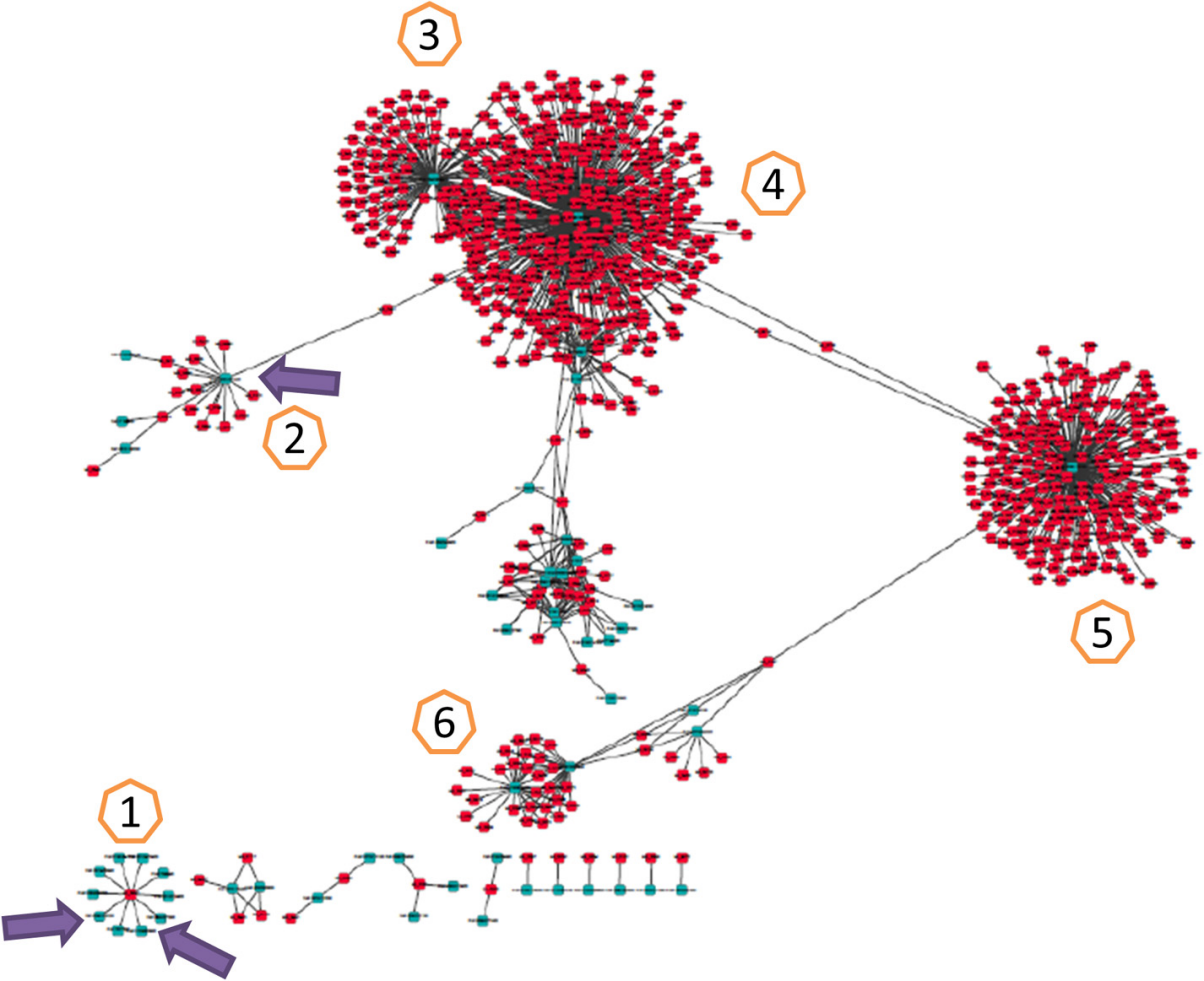
The independent network inferred from the top 1000 fungus-poplar relationships. Individual genes simultaneously connected with many other genes were sequentially marked as 1, 2, 3, 4, 5, and 6. Mark 1 represents a fungal metalloprotease gene that was surrounded by 10 poplar genes, including 2 DR genes; mark 2 is a poplar DR gene that is connected to 16 fungal genes. Marks 3 and 5 are the poplar genes secretory carrier 3 and NAD(P)-binding Rossmann-fold superfamily protein, respectively; marks 4 and 6 are poplar genes with unknown functions. The three arrows denote the position of 3 DR genes. The fungal genes are shown in red, and the poplar genes are shown in blue.

All F-P relationships were sorted based on relevance p-value in ascending order, and the top 1,000 (all P_relevance_ values were smaller than 10^−7^) were selected for network construction (Fig 5). The top 1,000 relationships contained 768 fungal genes and 54 poplar genes, of which 111 fungal genes and 36 poplar genes were shared with the preceding DEGs (S5 Fig). In the network, five poplar genes, one of which was a DR gene that related to 16 fungal genes (Fig 5, marker 2), were simultaneously connected to many fungal genes. In addition, of the 111 overlapping fungal DEGs, 64 genes related to the poplar gene Potri.008G120200 (Fig 5, marker 5), and most were up-regulated at the late stage in the 895/highly active group. Notably, only one fungal gene, a metalloprotease, was linked to many poplar genes (10 genes), which included 2 other DR genes. This fungal gene and 9 poplar genes around it also existed in the DEG list. The independent network highlighted the pivotal roles of DEG analysis and provided more information linking the host with the pathogen, thus displaying the critical events during the interaction.

### Effector prediction highlights a secreted metalloprotease

The effectors were often proteins secreted by the pathogen into host cells to enhance infection. Of the 10,040 fungal genes, 700 genes were predicted to produce secreted proteins, and 85 genes were DEGs. In the 895/highly active group, 16 genes were up-regulated at the early stage (12–48 h), including 1 metalloprotease, 1 Lys M domain gene, 2 pectate lyases, and 3 glycosyl hydrolases. In contrast, only 4 genes were up-regulated from 24–72 h in the 895/weakly active group. Of the 85 genes, 17 also existed in the 768 fungal gene list of top 1000 relationships. Intriguingly, only the metalloprotease gene was overexpressed at the early stage, whereas the three other genes, including D-isomer-specific 2-hydroxyacid dehydrogenase, copper-fist DNA-binding domain protein, and FAD-binding domain protein, were overexpressed at the late stage (48–96 h). Based on an effector-specific domain analysis, 12 genes were identified as candidate effectors. One hypothetical protein containing a DUF3129 domain (M6_09300) was significantly up-regulated at the early stage in the 895/weakly active and 895/highly active groups. In addition, in the top 1000 network, the M6_05038 gene was connected to 6 poplar genes, including plant L-ascorbate oxidase, which is a multifunctional molecule that supports plant growth and development [25]. Another two predicted effector genes were related to three poplar genes that included a galactosyltransferase family protein.

## Discussion

In this study, we performed a comprehensive expression profile analysis based on a time-course assay on the poplar-*M*. *brunnea* interaction using mixed samples, which provided for the accumulation of a large amount of data (42.6 Gbps).

Based on the profile analysis, we identified a number of infection-associated genes. The different appearances of highly and weakly active fungi provided another opportunity to inspect the mechanisms of the intrusion at the level of gene expression. Highly active fungi possess a powerful proliferation potential. During infection, they regulate glycosyl hydrolases and metalloproteases to degrade the host’s cell wall. Glycosyl hydrolases are extremely common enzymes with roles in nature that include the degradation of cellulose and hemicellulose, which are important components of the cell wall of a leaf[26]. Moreover, cellulose was degraded into glucose, providing sufficient nutrients to support the growth of the invader. In the 895/highly active group in our study, 10 sugar transporter genes were overexpressed, which suggested that the pathogen might utilize and compete for host nutrition.

Our preceding observations had demonstrated that many genes from the Lys M domain family were up-regulated in infected samples compared with uninfected leaves[17]. Chitin is widely present in fungal cell walls and can be recognized by the Lys M receptor in plants[27]; this recognition can trigger the PAMP-triggered immunity (PTI) response in host cells[1]. The LysM family of proteins function as effectors to suppress plant basal immunity during plant colonization, possibly through competitive combination with fungal chitin. In the 895/highly active group, the expression levels of the 4 Lys M family genes, as well as certain chitin-associated enzymes, were significantly altered (2 up-regulated, 2 down-regulated). These results again accentuate the importance of chitin in the pathogen-host interaction. Moreover, we did not find any sugar transporter, glycosyl hydrolases, metalloprotease, chitin, or LysM domain genes in the specific DEGs of the 895/weakly active group; however, certain Hsp proteins and senescence-associated proteins were found, which could explain the “weak” activity of these fungi. Intriguingly, the highly susceptible clone NL214 expressed none of these specific DEGs when infected with highly active fungi. These genes might be up-regulated at an early stage but show no significant changes from 48–96 h because the NL214 clone leaves lacked an efficient counterattack.

This dynamic transcriptome analysis revealed an intense rivalry at the pathogen and host gene expression levels in the 895/highly active group. Many critical fungal genes, especially certain well-known, infection-associated genes such as glycosyl hydrolases were found to be significantly regulated during infection. More poplar genes were up-regulated in the 895/highly active group at the early stage (from 12 to 48 h), and the number of up-regulated DE-DR genes was far greater than in the other two groups. Therefore, if the opponent is weak, the interaction between the pathogen and host would also show a low frequency.

The interaction between a host and pathogen is a complicated process. The DEG analysis only demonstrated the varied events during the infection, whereas network inference could determine the relationships between the changes in expression in the fungi and poplar and accurately framed the details of these interplays. That might be the reason why the overlapping gene between them only took a small proportion (S5 Fig). However, these “elites” would be good candidates in further functional research. In the top 1000 relationship network, the fungal DEG M6_08342 (metalloprotease) was connected to 10 poplar genes, including 2 DR genes. In fact, the pathological actions of metalloproteases have been widely investigated in human pathogenic microorganisms but scarcely investigated in plant pathogenic fungi [23,28]. However, in our study, the accumulated findings (DEGs analysis, network inference, and effector prediction) demonstrated that this metalloprotease gene might be a pivotal effector during infection and could be recognized by these two intracellular receptors (DR proteins). The most exciting findings were the five core poplar genes that were surrounded by many fungal genes. One of them was a disease-resistance gene, and none of the other four genes, including secretory carrier 3, NAD(P)-binding Rossmann-fold superfamily protein, and two hypothetical proteins, has been reported as associated with host defense. Despite their unknown functions, genes with high degrees (linked to many other genes) are also worthy of further investigation. Moreover, the well-known elicitor-activated gene ELI3 [29], which was down-regulated during the late stage, was linked to five fungal genes, especially the protein farnesyltransferase/geranylgeranyltransferase type I alpha subunit, which suggests an influence on signal transduction in the host-pathogen interaction. These relationships provide more reliable candidate genes for future functional validation.

The independent network implemented was suitable to join the fungal and poplar profiles because it exclusively considered the frequency of significant changes. Unlike the traditional relevance network[30,31], which often uses a correlation measure such as Pearson’s correlation coefficient, the independent network introduces ‘independence’ to reflect the association. This simple and non-parametric method will not be affected by the number of largely varied gene expressions, but their tendency, which may show better the core essence of the gene relationship. In addition, this strategy does not require strict time point sampling but works well with a flexible time course experiment, which is similar to the true environment and easier to apply. In our study, considering the shorter infection period of lowly active fungi and inappropriate T0, we tentatively chose three time points for each condition. The sample size is an important element in network analysis, and larger sample sizes typically yield better estimates [32]. More time points sampling in our assay would give a better performance. We are eager to assess the robustness of this network inference in future work, when more infected samples have been collected. It is not very rational to prove the relationship by just detecting the gene expression levels between different samples using realtime PCR. In addition, biological replicates of RNA-Seq are planned to ensure the reliability of our data and findings. It is recommended that the vital genes suggested in our findings should be given further validation, e.g. qPCR, before further study. Experiments such as gene knockout or knockdown (RNA silencing) will be performed to validate the confirmed candidate genes [33–35], which will also provide feedbacks for refinement of the network analysis algorithm.

In our independent network analysis, we excluded the gi(-)gj(-) condition to decrease the influence of inactive genes (e.g., stably expressed genes). In general, if the expression levels of genes A and B are not significantly altered in any of the observations, the relevance of the two genes should be high in the correlation analysis (e.g., Pearson’s correlation coefficient), which interferes with the assessment of the relevance between active genes. Consequently, we produced a corresponding test and improvements to address this problem. The limitation of this independent strategy is that the fourfold table does not have direction. For example, the 70/1/2/3 arrangement is completely equal to 3/1/2/70 and to 70/2/1/3. If the fourfold table was designed as A↑B↑/A-B↑/A↑B-/A-B-, we might expect a significant result, such as 70/1/2/3 (P<0.001), which means that A↑ is often accompanied by B↑. However, the 3/1/2/70 arrangement, which means that, in most cases, A and B show no significant alternation, also leads to a conclusion that the expression of gene A is highly correlated with that of gene B. Thus, in our study, we neglected the—cell and split the 3*3 grid into 4 fourfold tables (S1 Fig).

We compared our independent network with the widely used Pearson’s correlation analysis by inputting the same gene sets and expression values. All of the correlation coefficients between the fungal and poplar genes were calculated and sorted in descending order. The top 1000 relationships (R>0.9975) contained 102 fungal and 266 poplar genes and included 5 DR genes. However, all of these 5 DR genes showed low expression values in all samples, and the visualized network was quite scattered (data not shown). In contrast, the 3 DR genes in our network displayed higher expression values and were significantly regulated during infection. Furthermore, the web tool PlaNet (http://aranet.mpimp-golm.mpg.de/) was used to identify the co-expression of the 3 DR genes in our network [36]. However, only one DR gene (Potri.008G220200) could be found in this database, and its node vicinity network contained 55 co-expression genes that were significantly enriched in lipid and amino acid metabolism, signaling receptor kinases, and protein degradation.

## Conclusions

Our study provides a new host-pathogen profile investigation technology that has higher expression accuracy (RNA-seq, high-throughput) and higher time point accordance (simultaneous sequencing). A corresponding, large time course RNAseq dataset (42.6 Gbps) for poplar-fungi interactions is provided for further network inference and functional analyses. The simple and useful independent network strategy determined the relationships between fungus and poplar expression changes and accurately framed the details of these interplays. Taken together, we provide a clear framework of the interaction network and identify a number of candidate key effectors in this process, which might assist in functional tests, resistant clone selection, and disease control in the future.

## Supporting Information

S1 Fig Schematic diagram of P relevance calculation for the independent network analysis. (PDF)

S2 Fig The NL895 leaves infected with highly active fungi: (A) 96 h and (B) after 96 h. (PDF)

S3 Fig The P:M proportion between the 895/highly active and 895/weakly active groups. (PDF)

S4 Fig The detected genes between the 895/highly active and 895/weakly active groups. (PDF)

S5 Fig The top 1,000 relationships contained 768 fungi that shared several genes with the preceding DEGs. (PDF)

S1 Table Statistics for mapped reads and detected genes. (PDF)

S2 Table The distribution of DE-DR genes in sub-categories between the 895/highly active and 895/weakly active groups. (PDF)
