# Supplementary figures and images for: A Comprehensive Analysis of the Transcriptomes of Marssonina brunnea and Infected Poplar Leaves to Capture Vital Events in Host-Pathogen Interactions
Source: PLoS One. 2015 Jul 29;10(7):e0134246. doi: 10.1371/journal.pone.0134246 (PMC4519268; doi:10.1371/journal.pone.0134246)

S1 Fig.      Schematic diagram of P relevance calculation for the independent network analysis

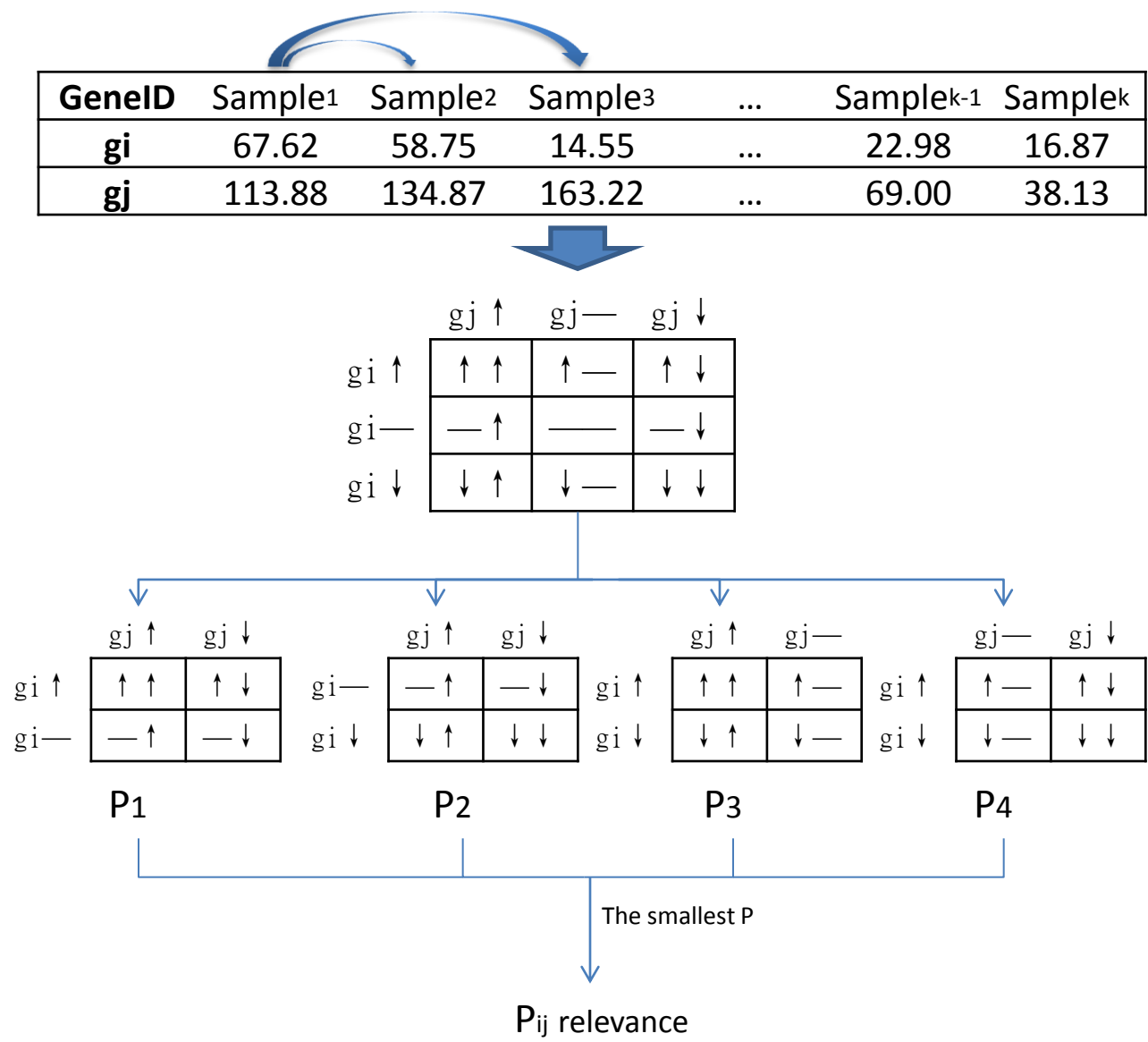

Supplement: S1 Fig — (PDF) [file pone.0134246.s001.pdf]

S2 Fig. The NL895 leaves infected with highly active fungi : (A) 96 h and (B) after 96 h

A

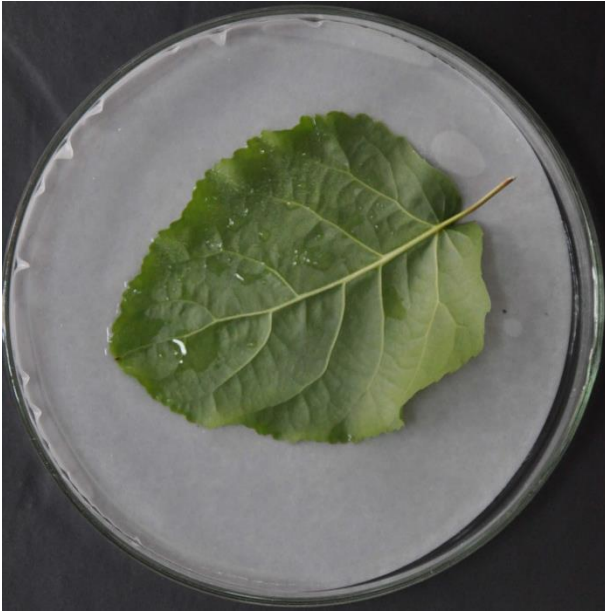

B

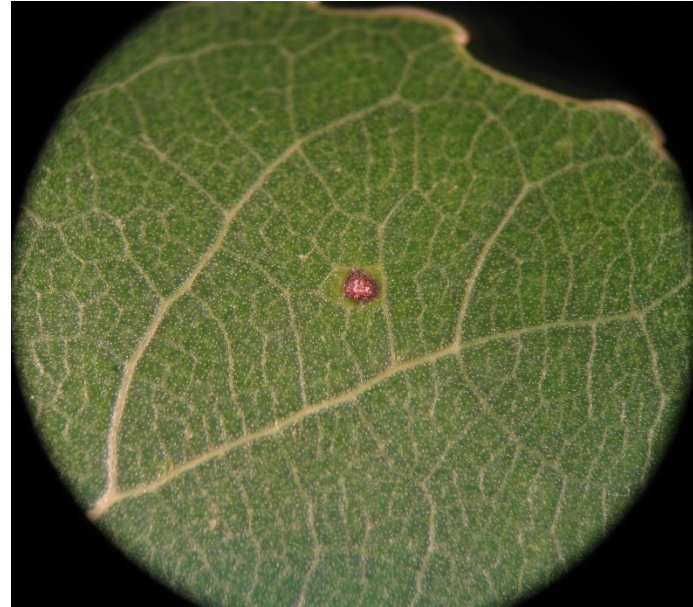

Supplement: S2 Fig — (PDF) [file pone.0134246.s002.pdf]

S3 Fig. The P:M proportion between the 895/highly active and 895/weakly active groups

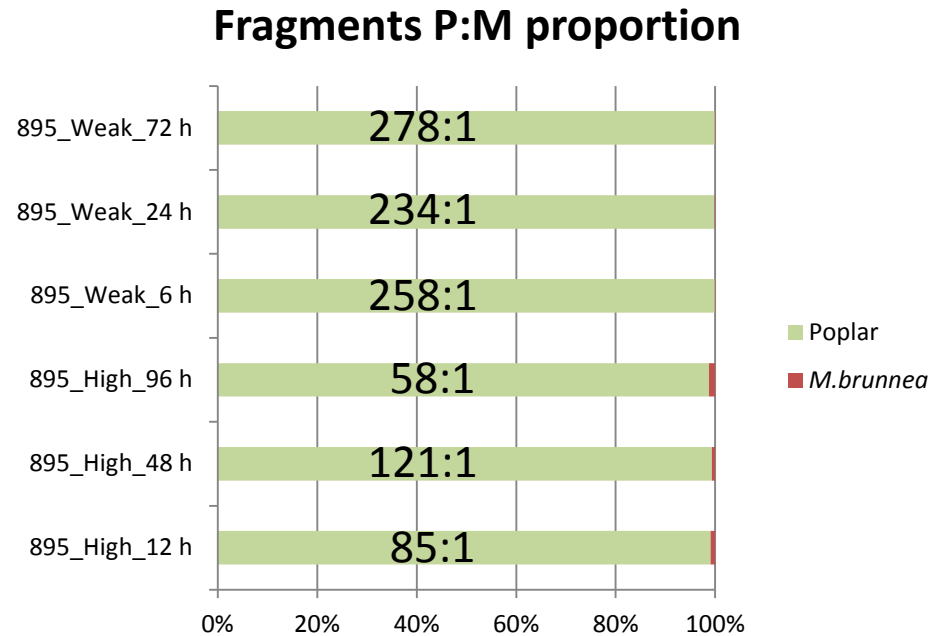

Supplement: S3 Fig — (PDF) [file pone.0134246.s003.pdf]

S4 Fig. The detected genes between the 895/highly active and 895/weakly active groups

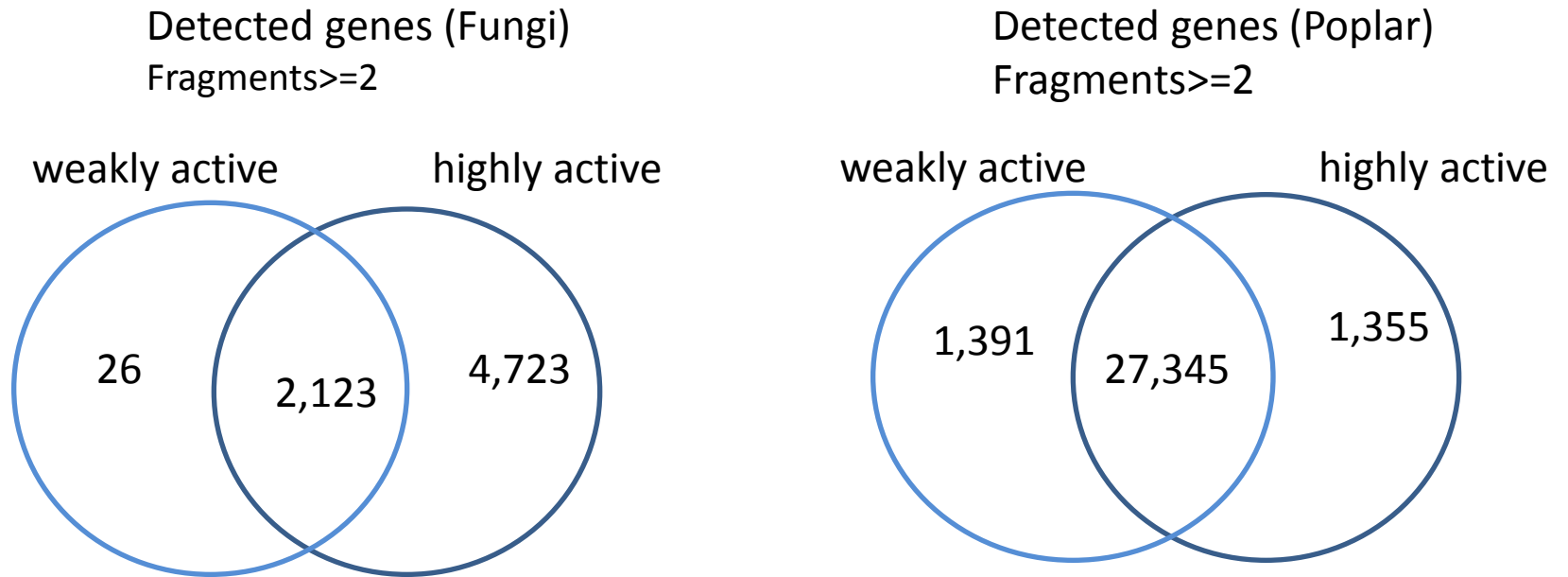

Supplement: S4 Fig — (PDF) [file pone.0134246.s004.pdf]
