# Supplementary material for: A Comprehensive Analysis of the Transcriptomes of Marssonina brunnea and Infected Poplar Leaves to Capture Vital Events in Host-Pathogen Interactions
Source: PLoS One. 2015 Jul 29;10(7):e0134246. doi: 10.1371/journal.pone.0134246 (PMC4519268; doi:10.1371/journal.pone.0134246)

S5 Fig. The top 1,000 relationships contained 768 fungi that shared several genes with the preceding DEGs

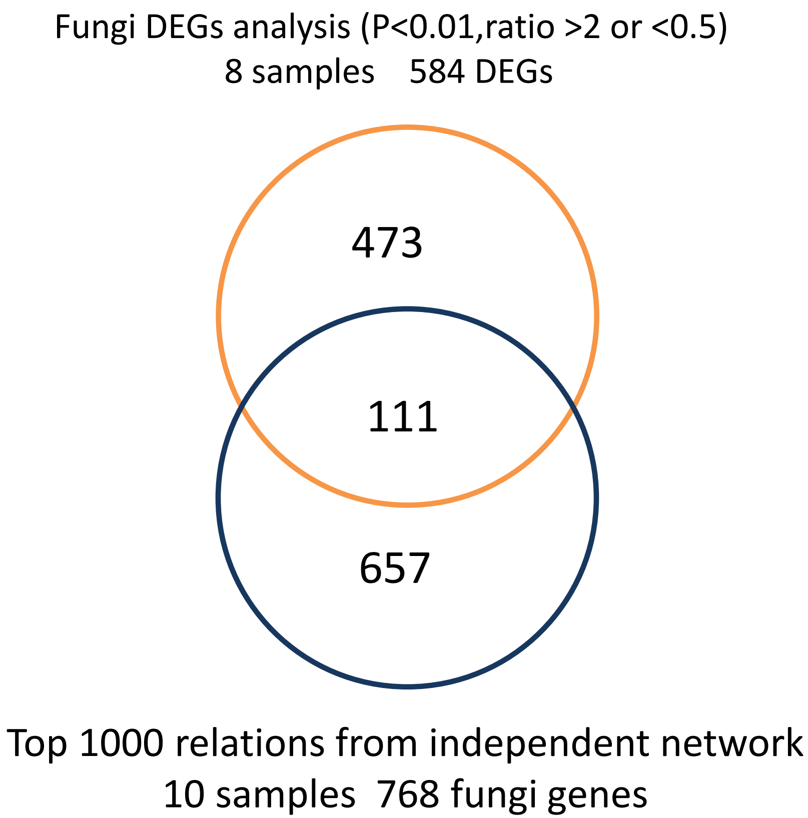

Supplement: S5 Fig — (PDF) [file pone.0134246.s005.pdf]
