# Supplementary material for: A Comprehensive Analysis of the Transcriptomes of Marssonina brunnea and Infected Poplar Leaves to Capture Vital Events in Host-Pathogen Interactions
Source: PLoS One. 2015 Jul 29;10(7):e0134246. doi: 10.1371/journal.pone.0134246 (PMC4519268; doi:10.1371/journal.pone.0134246)
Supplement: S1 Table — (PDF) [file pone.0134246.s006.pdf]

S1 Table. Statistics for mapped reads and detected genes.

	Raw Reads (million pairs)	Length (bp)	Data size (Gbps)	Mapped fragments (poplar)	Detected gene[a] (poplar)	Mapped fragments (fungi)	Detected genes (fungi)
**214_High_48 h[b]**	9.4	151	2.8	3,332,423	23,840	16,928	1,502
**214_High_96 h**	10.1	151	3.0	4,765,384	24,569	15,304	1,632
**214_Weak_24 h[c]**	22.7	100	4.5	13,089,007	25,596	13,339	1,087
**895_High_12 h**	14.4	100	2.9	10,014,506	26,474	92,717	5,250
**895_High_48 h**	15	100	3.0	10,738,751	26,220	69,963	5,026
**895_High_96 h**	17.8	100	3.6	11,333,846	26,003	145,303	6,148
**895_Weak_6 h**	13.1	100	2.6	9,743,550	27,040	11,504	1,338
**895_Weak_24 h**	11.5	100	2.3	8,071,463	26,250	10,666	1,281
**895_Weak_72 h**	12.8	100	2.6	8,716,076	25,177	5,347	649

[a] The criterion for "detected gene" was that the mapped fragments were not less than 2.

[b] "High" means infected with highly active fungi.

[c] "Weak" means infected with weakly active fungi.
