# Supplementary material for: A Comprehensive Analysis of the Transcriptomes of Marssonina brunnea and Infected Poplar Leaves to Capture Vital Events in Host-Pathogen Interactions
Source: PLoS One. 2015 Jul 29;10(7):e0134246. doi: 10.1371/journal.pone.0134246 (PMC4519268; doi:10.1371/journal.pone.0134246)
Supplement: S2 Table — (PDF) [file pone.0134246.s007.pdf]

**S2 Table. The distribution of DE-DR genes in sub-categories between the 895/highly active and 895/weakly active groups.**

| Disease-resistance protein | Total | 895/weakly active<br>6 h 24 h 72 h<br>U(D) [a] | 895/highly active<br>12 h 48 h 96 h<br>U(D) |
|---|---|---|---|
| NB-ARC domain-containing disease-resistance protein | 329 | 3(50) | 26(29) |
| Disease-resistance protein (TIR-NBS-LRR class) family | 143 | 0(44) | 10(8) |
| Disease-resistance family protein/LRR family protein | 18 | 0(2) | 2(0) |
| Disease-resistance protein (CC-NBS-LRR class) family | 33 | 0(8) | 2(4) |
| Disease-resistance protein (TIR class), putative | 2 | 0(2) | 0(1) |
| Disease-resistance protein (TIR-NBS class) | 13 | 0(1) | 1(0) |
| Disease-resistance protein (TIR-NBS class), putative | 3 | 0(1) | 0(0) |
| Disease-resistance protein (TIR-NBS-LRR class) | 13 | 0(4) | 2(0) |
| Disease-resistance protein (TIR-NBS-LRR class), putative | 68 | 0(16) | 2(3) |
| Disease-resistance-responsive (dirigent-like protein) family protein | 42 | 3(4) | 1(5) |
| DZC (Disease resistance/zinc finger/chromosome condensation-like region) domain-containing protein | 4 | 0(0) | 0(0) |
| ENHANCED DISEASE RESISTANCE 2 | 3 | 0(2) | 0(0) |
| LRR and NB-ARC domains-containing disease-resistance protein | 90 | 1(15) | 4(8) |
| Total | 761 | 8(148) | 50(58) |
| Total DEGs | | 10,375 | 7,089 |

[a]U, up-regulated; D, down-regulated
